# Case report: Sex-specific characteristics of epilepsy phenotypes associated with Xp22.31 deletion: a case report and review

**DOI:** 10.3389/fgene.2023.1025390

**Published:** 2023-06-06

**Authors:** Yi Wu, Dan Wu, Yulong Lan, Shaocong Lan, Duo Li, Zexin Zheng, Hongwu Wang, Lian Ma

**Affiliations:** ^1^Department of Pediatrics, Second Affiliated Hospital of Shantou University Medical College, Shantou, China; ^2^ Centre for Precision Health, School of Medical and Health Sciences, Edith Cowan University, Joondalup, WA, Australia; ^3^Department of Cardiology, Second Affiliated Hospital of Shantou University Medical College, Shantou, China; ^4^ Department of clinical Medicine, Guangdong Medical University, Zhanjiang, China; ^5^Department of Hematology and Oncology, Shenzhen Children’s Hospital of China Medical University, Shenzhen, China; ^6^ Shenzhen Public Service Platform of Molecular Medicine in Pediatric Hematology and Oncology, Shenzhen, China; ^7^ Department of Pediatrics, The Third Affiliated Hospital of Guangzhou Medical University (The Women and Children’s Hospital of Guangzhou Medical University), Guangzhou, China

**Keywords:** Xp22.31, epilepsy, microdeletion, copy number variations, X-linked ichthyosis, sex-specific

## Abstract

Deletion in the Xp22.31 region is increasingly suggested to be involved in the etiology of epilepsy. Little is known regarding the genomic and clinical delineations of X-linked epilepsy in the Chinese population or the sex-stratified difference in epilepsy characteristics associated with deletions in the Xp22.31 region. In this study, we reported two siblings with a 1.69 Mb maternally inherited microdeletion at Xp22.31 involving the genes *VCX3A, HDHD1, STS, VCX, VCX2*, and *PNPLA4* presenting with easily controlled focal epilepsy and language delay with mild ichthyosis in a Chinese family with a traceable 4-generation history of skin ichthyosis. Both brain magnetic resonance imaging results were normal, while EEG revealed epileptic abnormalities. We further performed an exhaustive literature search, documenting 25 patients with epilepsy with gene defects in Xp22.31, and summarized the epilepsy heterogeneities between sexes. Males harboring the Xp22.31 deletion mainly manifested with child-onset, easily controlled focal epilepsy accompanied by X-linked ichthyosis; the deletions were mostly X-linked recessive, with copy number variants (CNVs) in the classic region of deletion (863.38 kb–2 Mb). In contrast, epilepsy in females tended to be earlier-onset, and relatively refractory, with pathogenic CNV sizes varying over a larger range (859 kb–56.36 Mb); the alterations were infrequently inherited and almost combined with additional CNVs. A candidate region encompassing *STS*, *HDHD1*, and *MIR4767* was the likely pathogenic epilepsy-associated region. This study filled in the knowledge gap regarding the genomic and clinical delineations of X-linked recessive epilepsy in the Chinese population and extends the understanding of the sex-specific characteristics of Xp22.31 deletion in regard to epilepsy.

## Introduction

Epilepsy is a common neurological disturbance that manifests as recurrent and unprovoked seizures arising from largely unknown cellular and genetic mechanisms. Genetic contribution is increasingly emphasized in the etiology of epilepsies (Olson et al., 2014; EpiPM Consortium, 2015), notably copy number variants (CNVs) (Olson et al., 2014). Approximately 100 genes in CNV regions have been identified to be associated with epilepsy (EpiPM Consortium, 2015; Heyne et al., 2018), and rare sex chromosome CNVs have been identified in patients with epilepsy (Jabbari et al., 2018; Niestroj et al., 2020). However, evidence from case report studies has continuously suggested the involvement of Xp22.31 CNVs in epileptogenesis, for example, three case reports of X-linked familial focal epilepsy in the UK population (Mefford et al., 2011).

Although the specific causative genes for epilepsy and the functions of genes involved in this region are still elusive, CNVs in Xp22.31 have been suggested to play an important role in the genetics of epilepsy, albeit with variable penetrance (Mefford et al., 2011; Olson et al., 2014; Addis et al., 2018). Additionally, variants in the X chromosome lead to inconsistent disease phenotypes and prevalence rates between the sexes, owing to X-linked recessive inheritance and random X chromosome inactivation (XCI) in females, which protects females to a large extent (Fang et al., 2021). Furthermore, Xp22.3 is a domain of XCI escape and expresses genes such as STS with varying skewness, expanding the phenotypic diversity between the sexes (Meroni et al., 1996; Balaton and Brown, 2016; Balaton et al., 2018). Patients of both sexes with epilepsy have been found to have deletions in the Xp22.31 region (Olson et al., 2014; Addis et al., 2018; Gao et al., 2018); however, few studies have focused on the differences in variant size, source, and epilepsy patterns and types by sex.

Here, for the first time, we report two siblings with a maternally inherited microdeletion in a classic Xp22.31 region from a family with multifetal X-linked ichthyosis presenting with easily controlled focal epilepsy. Subsequently, we expanded the understanding of sex-specific characteristics of Xp22.31 deletion-associated epilepsy by reviewing the DECIPHER (Database of Chromosomal Imbalance and Phenotype in Humans Using Ensemble Resources) (Firth et al., 2009) and published articles. Together, we provided evidence for Xp22.31-microdeletion-related gene loss of function as an epilepsy risk factor using comparative genomic analysis.

### Case description

The probands were referred to the Pediatric Clinic at the Second Affiliated Hospital of Shantou University Medical College (SUMC). Informed written consent was obtained from the children’s mother. This study was approved by the Research Ethics Committee of the Second Affiliated Hospital of SUMC (2020-4).

### Proband 1

Proband 1 is currently 7 years and 8 months of age {height: 121.5 cm [−1.8 standard deviation (SD)], weight: 24.5 kg}. G1P1, at 38+ weeks of pregnancy, was born at term by C-section because of oligohydramnios detected by ultrasonic examination (birth weight: 2.8 kg). His mother noted no specific problems in the prenatal and perinatal periods. Intermittent daytime somnolence and nocturnal insomnia were problematic in infancy, but they returned to normal after age 3. The patient’s early developmental milestones were normal. His language development seemed delayed, as he could only say a few single words, e.g., “tea”,” mom”,” dad” at 1 year of age. He is presently in the first grade of primary school, and his academic performance is average.

**FIGURE 1:**
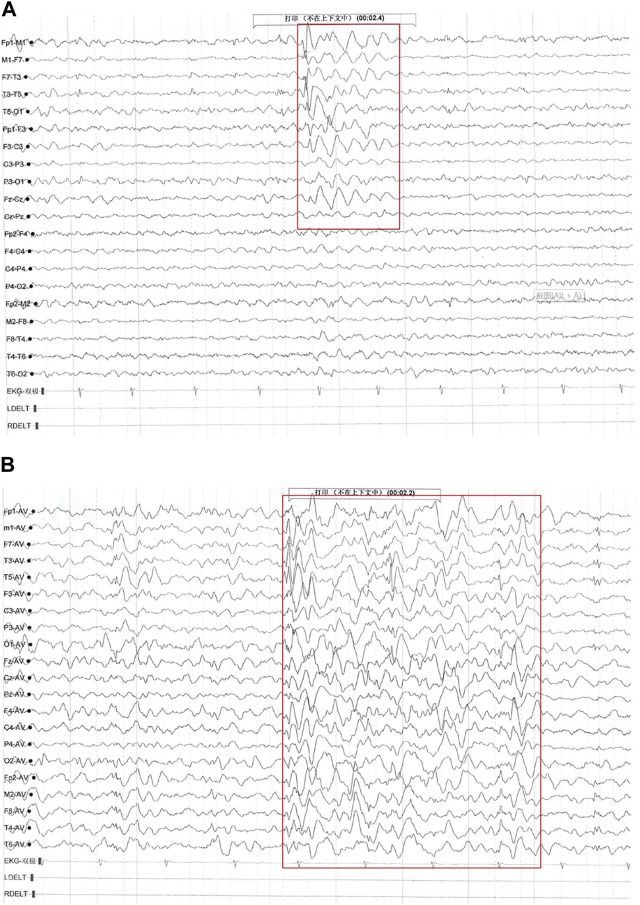
Electroencephalography (EEG) findings of Proband 1. Multiple paroxysmal events were recorded, with a clinical phenotype of sudden cessation of activity and daze. **(A)** During the interictal period, middle- and high-amplitude spine-slow waves were located in the bilateral temporal region, especially on the left side (EpiPM Consortium, 2015). **(B)** During the attack period, the low amplitude fast rhythm, obvious on the left, gradually evolved into a significant high amplitude spine-slow wave rhythm located in the bilateral temporal and frontal areas and Fz, and Cz showed and spread to the adjacent brain region (clinical representation: action stopped, daze).

The patient’s first seizure occurred at the age of 5^8/12^ years. He suddenly bowed, stared, and was unable to answer any questions while inside an elevator in the daytime. He recovered approximately 10 seconds later. Seizure attacks initially occurred every 3–4 days; later, seizures became more frequent, with almost 2-4 seizures daily. Each seizure lasted for ten to several tens of seconds, without notable provoking factors. The patient was admitted to the hospital and underwent 24-h electroencephalography (EEG), and abnormal brain waves were observed (Figure 1). No remarkable abnormality was found on brain magnetic resonance imaging (MRI). Griffiths Development Scales, Chinese (GDS-C) (Li et al., 2020) evaluated at the age of 7^8/12^ years suggested that his personal-social ability and language ability lagged behind those of the same age, with an overall developmental quotient (DQ) of 92. The patient was diagnosed with “epilepsy” and treated with “carbamazepine (CBZ)” (50 mg BID for a week and subsequently 100 mg BID). Seizures were subsequently completely controlled within 1 month, and later, he was regularly reviewed by EEG, with no significant seizure wave found. He has taken CBZ for more than 2 years and gradually reduced doses.

Whole exome sequencing (WES) at the ages of 7 years and 6 months identified a 1.69 Mb maternally inherited hemizygous deletion on the X chromosome short arm (chr X: 6,451,786-8,138,073), affecting eight genes: *VCX3A* (OMIM 300533)*, HDHD1* (OMIM 306480)*, STS* (OMIM 300747)*, VCX* (OMIM 300229)*, PNPLA4* (OMIM 300102), *MIR4767, MIR4767*, and *RPS27AP17*. Details regarding WES were as follows: sheared blood DNA was prepared for Illumina paired-end sequencing and enriched for target regions with Agilent’s SureSelect Human All Exon V6 capture technology. The exome-captured library preparation was sequenced with the NovaSeq 6000 platform, reaching an average depth of >100X and Q30 > 90%. Reads were aligned to the GRCh37 human reference genome with BWA (v0.5.10). GATK Haplotype Caller v3.4 was used for variant calling and recalibration.]

### Proband 2

Proband 2 is the youngest brother in this family. He is currently 3^10/12^ years old. The birth history demonstrated no obvious special characteristics. No apparent feeding difficulties or sleep disorders were seen during infancy. Early milestones were mostly within normal limits.

His first seizure occurred at 3^8/12^ years. In the morning, he was scolded by his father and in a bad mood. Approximately 10 minutes later, he suddenly tilted his head to one side, with his eyes open and turning upward, and could not talk or answer questions. The seizure lasted approximately half an hour; afterward, the patient became very drowsy and did not want to talk. A similar event occurred 1 hour later in the emergency room of a hospital, lasting for 15 min. Then, the patient was admitted to a pediatric department. A third event occurred 3 days later, with an approximately 3-min gaze described by a pediatrician. His body temperature was normal at the time, and he showed no signs of intercurrent illness.

The patient underwent a 5-h video-EEG during hospitalization, and no seizures occurred during the EEG test. The EEG showed that the background activity was normal, and many sharp waves were emitted in the left central area (only EEG report available). No obvious abnormality was found on the brain MRI. The patient was diagnosed with epilepsy and treated with valproic acid (VPA) (first 65 mg BID for 3 days and gradually increasing the dosage in accordance with the standard protocol, currently 130 mg BID). After taking VPA for 10 days, no similar symptoms were observed and have remained absent thus far. The EEG reexamined every 2 months thereafter was normal. Mild developmental and speech delays were also reported by a physiotherapist and a pediatrician, with GDS-C at the age of 3^10/12^ years showing that his hearing and language and practical reasoning abilities lagged behind his peers; the total DQ score was 84. At this current age, the proband displayed moderate failure to thrive; height: 94 cm (−2 SD) and weight: 13 kg (−2 SD).

WES on blood DNA found the same maternally inherited deletion at Xp22.31 found in his elder brother.

### Skin problems

**FIGURE 2:**
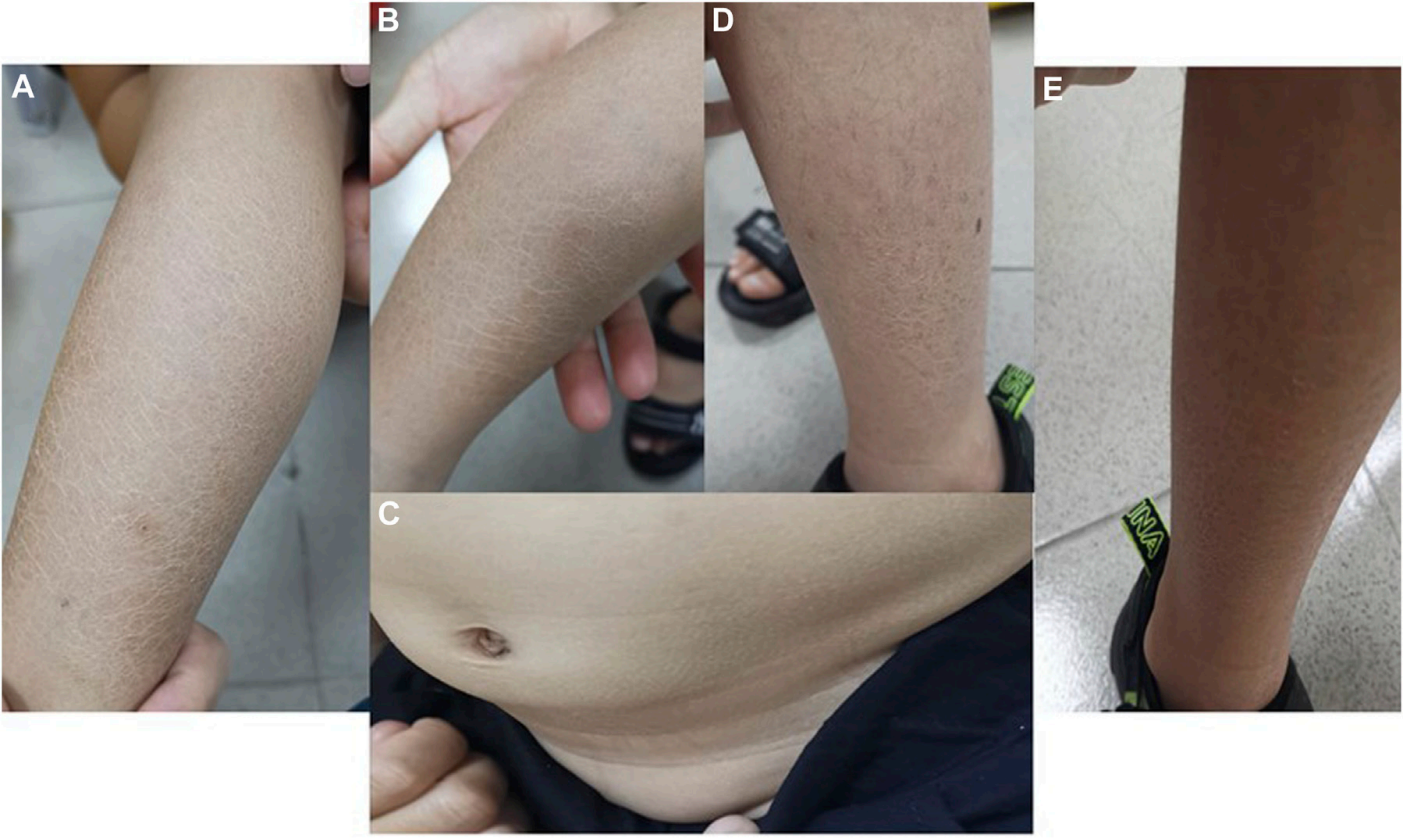
Photographs of ichthyosis on the skin **(A, B)** Photos of both lower limbs of Proband 1, with obviously dry and scaly skin in summer; **(C)** abdominal skin of Proband 1; **(D, E)** lateral skin of both lower limbs of Proband 2, with dry and scaly skin but no ulcer or break in summer.

Both boys had blatant ichthyosis since their early infancy. The abdominal wall and extremities showed epidermal dryness and scaling skin even in summer, consistent with ichthyosis (Figure 2), and deterioration was seen in cold weather or winter. However, the skin lesions did not appear severe when applying daily moisturizer to improve their symptoms.

### Other Xp22.31 deletion-related comorbidities

No other comorbidities, including cryptorchidism, corneal opacity or cardiac arrhythmia, were noted in the two children after a detailed physical examination by a specialist. Nor were the apparent clinical presentations of ADHD and autism, despite a lack of professional scale evaluations for ADHD or autism for both children.

### Family history

Both probands’ parents are of pure Chinese descent. The mother was 36 years old (height: 153 cm; weight: 51 kg), and the father was 40 years old (height: 170 cm; weight: 62 kg). Neither parent experienced epilepsy, skin abnormalities or other neurodevelopmental disorders. WES showed that their mother was an Xp22.31 microdeletion carrier, and their father did not carry any exon abnormalities.

The probands have a sister, the second child in the family, currently 6 years old. Her birth history and early developmental milestones were normal. Unlike her two brothers, her skin was smooth and delicate and good at communication. She was free of symptoms of epilepsy, speech delay and other neurological abnormalities. WES revealed her to be a carrier of the Xp22.31 microdeletion.

**FIGURE 3:**
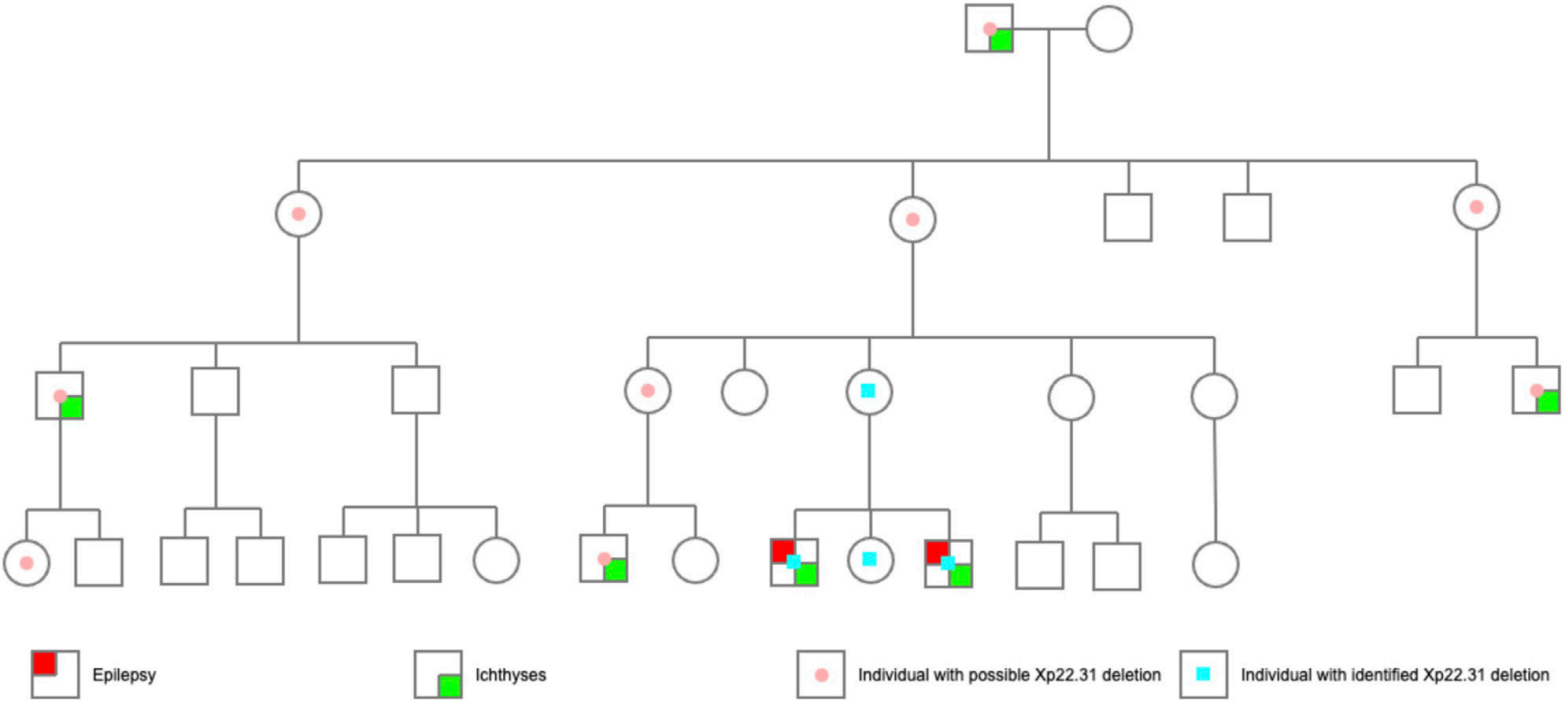
The pedigree of the probands. The probands’ great grandfather had obvious skin ichthyosis, whereas their great grandmother had normal skin; the second generation (their great grandfather’s 6 children) did not have the ichthyosis phenotype, but the males (the third generation) born to the women in this generation manifested a noticeable skin ichthyosis phenotype. Of the 10 children in the fourth generation, only three boys born to women in the third generation had an ichthyosis phenotype. Proband 1 and Proband 2 had epilepsy. Another boy, currently 2 years old, had no seizure attack thus far.

**FIGURE 4:**
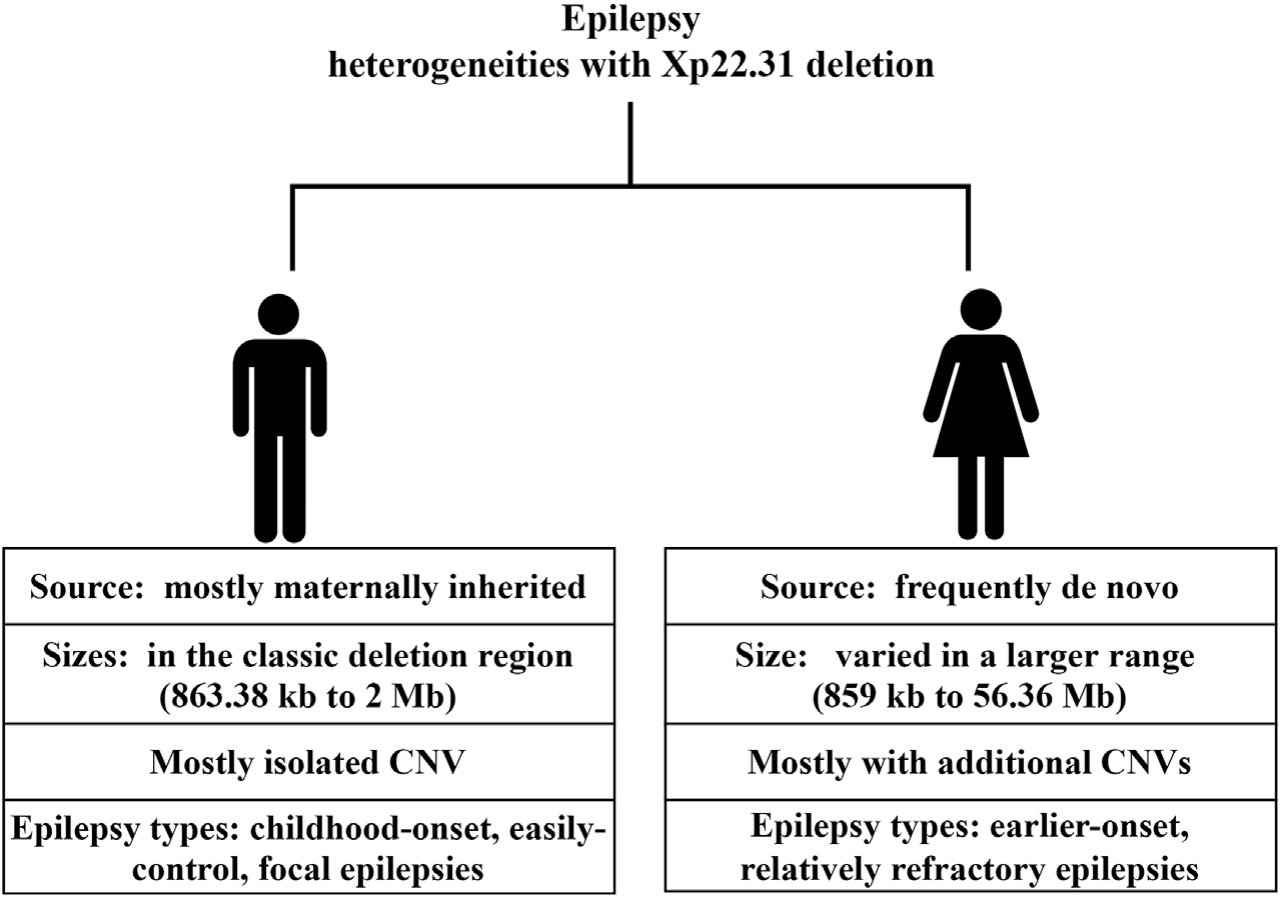
Summary of sex-specific epilepsy heterogeneities with Xp22.31 deletion.

A maternal family history of epilepsy or intellectual disability (ID) was denied by the proband’s mother. However, ichthyosis has been present since the great grandfather’s generation (Figures 3, 4). A total of 6 males in 4 generations of the family manifested with ichthyosis; however, only the two probands additionally manifested with epilepsy.

Unfortunately, a detailed medical history collection, systematic examination or genetic testing of the rest of the family is unlikely, as another family adopted her mother at age ten, and she gradually lost contact with no desire to reconnect.

### Characteristics of the reported epilepsy phenotypes with deletion in Xp22.31

We further searched the published studies and data from the DECIPHER database until Feb. 2022 and documented 23 patients with epilepsy and overlapping deletions in the Xp22.31 region ranging in size from 859 kb to 155.71 Mb (Supplementary eTable S2). Together with our 2 probands, there were 25 patients available for further examination. Of those, 14 were male, 8 were female, and 3 were of unknown sex. With regard to sex differences in variant sources, males were mostly maternally inherited (12/14), without any confirmed *de novo* change; in females, 4 were *de novo* (4/8), 2 were inherited (2/8), and the remaining 2 were of uncertain inheritance. The deletion sizes also differed greatly by sex. The majority (13/14) of males had isolated genetic changes, ranging from 863.38 kb to 2 Mb (mostly residing in the classic Xp22.31 deletion region). In contrast, females harbored deletion sizes that varied on a large scale (859 kb–56.36 Mb); three of them had isolated CNVs, and the rest (5/8) had additional CNVs in other chromosomes. Regarding the sex-associated epilepsy types, males tended to have focal or general epilepsy that was easily controlled with no or monotherapy with antiepileptic drugs. While data available for examining therapeutic effects were limited, females with Xp22.31 deletion tended to have early-onset and refractory epilepsy. Of 5 females with available data on seizure age or type (5/8), except for one patient with a *de novo* deletion of 1,688 kb at Xp22.31 who manifested with Rolandic epilepsy at age 6, the rest (4/8) had seizure types including infantile spasm (2/4) and generalized tonic-clonic seizures (GTC) (2/4). The epilepsy onset age was earlier in females than in males, with the earliest age of onset being 2 weeks. Notably, all 4 females with early-onset and refractory epilepsy harbored additional CNVs beyond the Xp22.31 deletion (4/4). Skin ichthyosis was notable in most of the included males rather than females. Other neurodevelopmental comorbidities associated with the Xp22.31 deletion included ID; among 14 males, 2 had uncertain status, 4 presented with ID, and the rest (8/14) had normal intelligence. In contrast, 4 females out of 8 had ID phenotypes.

CNVs in the deficit region of Xp22.31 of our probands encompassed genes including *VCX3A, HDHD1, STS, VCX, PNPLA4, MIR4767, MIR651* and *RPS27AP17* (Supplementary eTable S2). The minimum deletion region was 859 kb in females, though an additional 452 kb deletion in the Xq22.1 region and 33 kb deletion in 2p11.2 may confound the influences on epilepsy. In males, the minimum deletion region was 863.38 kb. The candidate region in Xp22.31, overlapping with the potentially pathogenic regions in all epilepsy cases, comprising *STS*, *HDHD1*, *and MIR4767*, is a risk factor for seizures.

## Discussion

For the first time, we described two Chinese sibling boys with a maternally inherited deletion at Xp22.31, presenting with easily controlled focal epilepsy and mild ichthyosis from a family with a long history of XLI. Additional abnormalities included language delay in both probands and weak practical reasoning ability and growth retardation in Proband 2. Both probands possessed an ∼1.6 Mb microdeletion at Xp22.31. We further summarized the sex-specific differences in epilepsy characteristics in Xp22.31 deletion, focusing on variant source, size and epilepsy types, onset age and therapeutic experience, which are clinically informative. Furthermore, a candidate region encompassing three genes, *STS, HDHD1*, and *MIR4767*, should be the likely pathogenic epilepsy-associated region, despite a failure to further narrow down the region compared to Meyers’s study (2020) (Myers et al., 2020).

Consistent with our findings in the Chinese population, Xp22.31-linked epilepsy associated with XLI in other ethnicities/races manifested as an easily controlled epilepsy phenotype and normal brain MRI findings (Mefford et al., 2011; Olson et al., 2014; Myers et al., 2020). Interestingly, despite a long history of notable ichthyosis, no members of this family experienced seizure attacks other than the reported probands. We have several conjectures. First, X-linked recessive epilepsy likely displays incomplete penetrance (Mefford et al., 2011), as a minority (13%) of boys with CNVs in the most common disrupted regions had epilepsy with XLI (Rodrigo-Nicolás et al., 2018). A few literature reports and cohort studies have collectively implied that recurrent variants at the Xp22.31 region conferred risk for epilepsy but with variable penetrance (Olson et al., 2014; Addis et al., 2018; Choi et al., 2020). Second, it is possible that the size of CNVs in the same chromosomal region in the same family is not stable throughout inheritance. The mother may have inherited the *STS* deletion from her mother and her maternal grandfather, and then again, a new nonallelic homologous recombination (NAHR) event occurred in oogenesis to extend the *STS* deletion to the *VCX* gene (Van Esch et al., 2005). In Khelifa’s study, the size of the deletion in the healthy-phenotype mother differed from that in her son (Ben Khelifa et al., 2013). Mencarelli et al. also revealed an additional duplication involving the genes *VCX*, *PNPLA4*, and *VCX2* at Xp22.3 in the proband relative to the mother (Mencarelli et al., 2008). Additionally, we could not completely exclude the involvement of environmental factors (e.g., toxin exposures during pregnancy) in epileptogenesis (Fukata and Fukata, 2017) that accounted for the occasional episode of seizures in the family clan.

It is essential to point out that current epilepsy–CNV associations have been identified through candidate locus screens. Accurate and efficient clinical consultation depends on the accumulation of clinical data. Although a male predominance in epilepsy phenotypes associated with Xp22.31 deletion was suggested in case reports (Rodrigo-Nicolás et al., 2018), we noted that both sexes with a deletion in Xp22.31 show epilepsy phenotypes. However, the types and severity of epilepsy and the source and size of variants differed greatly between sexes. Males who possess the Xp22.31 deletion mostly manifested with child-onset, easily controlled focal epilepsies that were X-linked recessive, accompanied by XLI. In contrast, except for one Rolandic epilepsy onset at age 6 years harboring a *de novo* deletion of 1,688 kb at Xp22.31 (7), epilepsy in females tended to be earlier-onset, relatively refractory and poor-prognosis, e.g., infantile spasm (Lux, 2013; Sahu, 2014) and GTC (Bresnahan et al., 2020), with pathogenic CNVs that were less likely to be inherited and combined with additional CNVs (4/4). The *de novo* variants and the combined additional CNVs may collectively account for the severity of seizure types. One Chinese girl with a *de novo* microdeletion at these same loci involving 4 genes (*STS, VCX, PNPLA4*) manifested a severe epilepsy phenotype (infantile spasms and hypotonic) and severe ID (Gao et al., 2018). *De novo* aberrations are known to cause worse outcomes than inherited changes (Watson et al., 2014). Previous findings have demonstrated that disease-causing *de novo* variants were associated with epilepsy types with the poorest prognosis (developmental and epileptic encephalopathies) (Allen et al., 2013). Additionally, we observed diverse combined neurodevelopmental abnormality phenotypes with epilepsy in Xp22.31 deletion in the review. The diverse manifestations of the aforementioned epilepsy phenotype, ID and speech delay are likely to support the incomplete penetrance property. Furthermore, the majority (8/13) of the males with CNV deletions from breakpoint *STS* and *VCX-B* manifested with focal epilepsy and ichthyosis but had normal cognition (Doherty et al., 2003), despite a deletion of a purported ID gene (*VCX-3A*). This finding supports mounting evidence that *VCX-A* is not an ID-associated gene (Cuevas-Covarrubias and González-Huerta, 2008; Mochel et al., 2008).

Notably, inherited deletions in Xp22.31 mostly contributed to epilepsy in males, whereas *de novo* deletions in Xp22.31 associated with severe epilepsy were observed only in females. Differing genetic backgrounds likely play a role in the contrasting enrichment patterns. We interpreted the lower susceptibility of males to complex refractory epilepsy as follows: *de novo* deletions in Xp22.31 are always lethal for males, and inherited deletions in Xp22.31 resulted in milder and weaker effects on females than on males. This finding is consistent with the phenomenon that some females, but not all, with the same X-linked deleterious allele are protected from its effects (Migeon, 2020).

There are several explanations underlying the diverse characteristics between sexes and among individuals. Lacking a second compensatory allele, males are naturally more frequently affected or have more severe phenotypes by X-linked pathogenic variants than females (Lyon, 1961). Phenotypes in females will be slightly milder than those in males, as females potentially have half the dose of pathogenic gene expression. Additionally, skewed XCI and XCI escape in the Xp22.31 region play an important role in the phenotypic heterogeneity of X-linked disorders (Tukiainen et al., 2017; Fang et al., 2021). Human genes that escape X inactivation are expressed at different levels in different individuals and in different tissues, as well as in different cells (Brown and Greally, 2003; Carrel and Willard, 2005; Migeon, 2020), suggesting remarkable expression heterogeneity among females (Zhang et al., 2013). As such, in females who possess the Xp22.31 deletion, the manifestation of phenotypes depends on whether the mutation is dominant, the expression level from the inactive X and the level of XCI skewing (Zhao et al., 2021). Indeed, skewed X-inactivation patterns due to X-chromosomal rearrangements have been reported to affect phenotypes of Xp22.3 defects (Suzuki et al., 2016; Ogushi et al., 2019). Additionally, the combined additional CNVs may contribute to the manifestation of phenotypes. Another possibility is ascertainment bias, whereby males with Xp22.31 deletions are picked up earlier than females due to their ichthyosis (only the most severely affected deletion-carrying females are likely to present), which may partly underlie the sex-specific phenotypic difference.

Moreover, it is important to note that the phenotypes may be immature and gradually develop during childhood. Males may have increased long-term risks of atrial fibrillation, leukemia, and end-stage renal disease, which need attention (Brcic et al., 2020). Females who possess genetic mutations or deletions in the Xp22.31 regions should be monitored for abnormal behavioral and psychiatric phenotypes (Cavenagh et al., 2019). Although the mother and her daughter appeared to be healthy carriers of this deletion, psychological evaluation is still needed, especially in the *postpartum* period (Cavenagh et al., 2019).

Genetic characterization of deleted and remaining sequences is an efficient way to map gene locations and functions associated with specific diseases (Monaco et al., 1985). Of the likely pathogenic genes, *STS* was supposed to be closely associated with the presence of epilepsy (Myers et al., 2020), as *STS* encodes a steroid sulfatase that hydrolyses neuro steroids that affect membrane potential and current conductance of the neuron, potentially controlling network excitability and seizure susceptibility (Ballabio et al., 1989a; Alperin and Shapiro, 1997; Kříž et al., 2008). Nevertheless, we must also acknowledge that *STS* deficiency alone is insufficient to cause seizures and that additional genetic or otherwise risk factors may be involved in the development of epilepsy. Additionally, *HDHD1*, encoding a pseudouridine-5′-phosphatase expressed at higher levels in the human brain, is also likely a possible gene candidate responsible for the seizure phenotype (Jenkins et al., 2004; Myers et al., 2020), as most of the epilepsy cases with Xp22.31 deletions harbored an *HDHD1* deficiency in addition to *STS* (Supplementary eTable S2). Notably, the clinical abnormalities associated with Xp22.31 CNVs are thought to be influenced by contiguous gene syndromes, which manifest complex phenotypes resulting from the codeletion of adjacent genes (Ballabio et al., 1989b; Trent, 1993). Thus, further cytogenetic exploration to unravel not only the specific gene functions but also the interactions between genes and resulting gene function changes is warranted.

The limitations of this study should be noted. While we performed an exhaustive literature search on prior studies and the Decipher Database, the case review on epilepsy associated with Xp22.31 deletion is not all-inclusive. It is, however, the most comprehensive study thus far and provides enough data to highlight the variable epilepsy characteristics based on variant source, size, seizure type, onset age, and therapeutic effect between sexes. Additionally, we could not obtain the genetic background of the other members in the probands’ family despite the long family history of notable ichthyosis.

## Conclusion

Our study demonstrates the significance of deletion at Xp22.31 in epilepsy and enriches the clinical phenotype profiles pertaining to the Xp22.31 microdeletion. Further investigation of the Xp22.31 region is warranted to unravel the pathophysiology of neurodevelopmental disorders.

## Data Availability

The datasets used and analyzed during the current study are available from the corresponding authors on reasonable request. Requests to access the datasets should be directed to HW, 458213437@qq.com.
