# Supplementary material for: Case report: Sex-specific characteristics of epilepsy phenotypes associated with Xp22.31 deletion: a case report and review
Source: Front Genet. 2023 Jun 6;14:1025390. doi: 10.3389/fgene.2023.1025390 (PMC10280017; doi:10.3389/fgene.2023.1025390)
Supplement: Supplementary file 1 [file Table1.docx]

Supplementary Material

**eTable 1** Clinical features of patients with epilepsy in Xp22.31 microdeletions

**eTable 2** Genes involved in chr X: 6451786-8138073 (hg19) microdeletion

**eTable 1** Clinical features of patients with epilepsy in Xp22.31 microdeletions

| Clinical features | Sex | Nationality | Seizure onset age | Seizure type | Drugs | XLI | ID/DD | ASD | SD | Site | Size | Genes | Inheritance | Other genetic disorders |
| --- | --- | --- | --- | --- | --- | --- | --- | --- | --- | --- | --- | --- | --- | --- |
| Proband 1 | M | Chinese | 5^8/12^ Y | Focal | CBZ | + |  | - | + | X: 6,451,786-8,138,073 | 1.6 Mb | *VCX3A*，STS, *HDHD1*， *PNPLA4,VCX,MIR4767,MIR651, RPS27AP17* | Maternally inherited | - |
| Proband 2 | M | Chinese | 3^8/12^ Y | Focal | VPA | + |  | - | + | X: 6,451,786-8,138,073 | 1.6 Mb | *VCX3A*， *STS, HDHD1*， *PNPLA4,VCX,MIR4767, MIR651, RPS27AP17* | Maternally inherited | - |
| Khelifa 2013[1] | M | Tunisia | 7 Y | Convulsions with myoclonic seizures | VPA | + | + | - | - | X: 6,395,312-8,383,288 | 2 Mb | *VCX3A*, *HDHD1*, *STS, VCX, VCX2, PNPLA4, MIR4767, MIR651,PRS5P8 RPS27AP17* | Maternally inherited | - |
| Doherty 2008 pt1 [2] | M | Sweden and German | 8 Y | Generalized Epilepsy | VPA | + | - | - | - | break point between STS and VCX–B | NA | *STS*，*SHOX*, *DXYS129*,  *VCX*, *PNPLA4* | Maternally inherited | - |
| Doherty pt2 [2] | M | Sweden and German | 11 Y | Generalized Epilepsy | VPA | + | - | - | - | break point between STS and VCX–B | NA | *STS*，*SHOX*, *DXYS129*, *VCX*, *PNPLA4* | Maternally inherited | - |
| Myers 2020 [3] | M | Canada | 8 Y | Focal, CECS | LEV | + | - | - | - | X: 6,598,822-7,500,757 | 902 kb | *HDHD1*, *STS,* *MIR4767, RPS27AP17* | Maternally inherited | - |

**eTable 1** Clinical features of patients with epilepsy in Xp22.31 microdeletions (continued)

| Clinical features | Sex | Nationality | Seizure onset age | Seizure type | Drugs | XLI | ID/DD | ASD | SD | Site | Size | Genes | Inheritance | Other genetic disorders |
| --- | --- | --- | --- | --- | --- | --- | --- | --- | --- | --- | --- | --- | --- | --- |
| Myers 2020 [3] | M | Canada | 7 Y | Focal | LEV | + | - | - | - | X: 6,598,822-7,500,757 | 902 kb | *HDHD1*, *STS,* *MIR4767, RPS27AP17* | Maternally inherited | - |
| Myers 2020 [3] | M | Canada | 10 Y | Focal | - | + | - | - | - | X: 6,456,777-8,119,329 | 1.66 Mb | *HDHD1*, *STS*, *VCX*, *PNPLA4, VCX3A,* *MIR4767, RPS27AP17* | Maternally inherited | - |
| 1719 | M | N/A | ≤29 Y | N/a | N/A | + | + | N/A | N/A | X: 6,628,264-7,491,648 | 863.38 kb | *HDHD1*, *STS,* *MIR4767, RPS27AP17* | Unknown | - |
| 276056 | M | N/A | ≤4 Y | Focal | N/A | + | - | - | + | X: 6,533,660-8,216,202 | 1.68 Mb | *VCX3A*, *HDHD1*, *STS*, *VCX*, *PNPLA4*, *VCX2,* *MIR4767, MIR651, RPS27AP17* | Maternally inherited | - |
| 283802 | M | N/A | ≤12 Y | N/A | N/A | + | + | - | - | X: 6,446,509-8,128,749 | 1.63MB | *VCX3A*, *HDHD1*, *STS*, *VCX*, *PNPLA4*, *VCX2* *MIR4767, MIR651, RPS27AP17* | Maternal inherited | Chr 2: 148,815,343-148,858,581 (del) |
| Deletion 2 [4] | M | N/A | 5 Y | N/A | N/A | N/A | N/A | N/A | N/A | X: 6,477,006-  8,091,810 | 1.6 Mb | *VCX3A*, *HDHD1*, *STS*, *VCX*, *PNPLA4*, *VCX2* *MIR4767, RPS27AP17* | Unknown | - |

**eTable 1** Clinical features of patients with epilepsy in Xp22.31 microdeletions (continued)

| Clinical features | Sex | Nationality | Seizure onset age | Seizure type | Drugs | XLI | ID/DD | ASD | SD | Site | Size | Genes | Inheritance | Other genetic disorders |
| --- | --- | --- | --- | --- | --- | --- | --- | --- | --- | --- | --- | --- | --- | --- |
| Malik [5] | M | Saudi Arabian | 11 Y | Complex, focal seizure | Carbamazepine and lorazepam | + | + | N/A | + | X: 6,456,036-8,139,238 | 1.68 Mb | *VCX3A, HDHD1,*  *VCX, STS, PNPLA4,* *MIR4767, RPS27AP17* | Maternally inherited | - |
| Deletion 3 [4] | M | N/A | 4 Y | Focal RE | N/A | N/A | N/A |  |  | X: 6,467,403- 8,091,751 | 1.6 Mb | *VCX3A, HDHD1*, *STS*, *VCX*, *PNPLA4*, *MIR4767, RPS27AP17* | Unknown | - |
| K. Gao [6] | F | Chinese | 4 Y | Infantile spasms | - | N/A | + | - | - | X: 6,705,268- 7,942,835 | 1,24 Mb | *STS*, *VCX*, *PNPLA4,* *HDHD1,* *MIR4767, RPS27AP17* | De novo | 5q13.2: 1.77 Mb (del) |
| Laura addis [7] | F | Sardinian | 6 Y | AE, RE | N/A | N/A | + | + | + | X: 6,449,682-8,138,035 | 1.6 Mb | *VCX3A*, *HDHD1*, *STS*, *VCX*, *PNPLA4*, *VCX2, MIR4767,MIR651, RPS27AP17* | De novo | - |
| Deletion 1 [4] | F | N/A | 2 W | Focal, infantile seizures | N/A | N/A | N/A | N/A | N/A | X: 7,061,905- 7,920,059 | 859 kb | *HDHD1 STS, PNPLA4, VCX, MIR4767* | De novo | Xq22.1: 452 kb, (del) 2p11.2: 33 kb (del) |
| Deletion 4 [4] | F | N/A | 11 M | Focal, GTC | N/A | N/A | N/A | N/A | N/A | X: 2,710,316-12,342,188 | 9.6 Mb | 66 genes | Presumed de novo | Xp22.2- q28:142 Mb (dup) |

**eTable 1** Clinical features of patients with epilepsy in Xp22.31 microdeletions (continued)

| Clinical features | Sex | Nationality | Seizure onset age | Seizure type | Drugs | XLI | ID/DD | ASD | SD | Site | Size | Genes | Inheritance | Other genetic disorders |
| --- | --- | --- | --- | --- | --- | --- | --- | --- | --- | --- | --- | --- | --- | --- |
| 256671 | F | N/A | <1 Y | Generalized tonic seizure | N/A | - | - | - | - | X: 6,545,045-8,176,172 | 1.63 MB | *HDHD1*, *STS*, *VCX*, *PNPLA4*, *VCX2, MIR4767, MIR651,* *RPS27AP17* | Paternal inherited | Chr 2: 50,574,095-50,940,737  (del) |
| 288318 | F | N/A | N/A | N/A | N/A | - | - | - | - | X: 10,701-56,368,981 | 56.36 Mb | 665 genes | Unknown heterozygous | X: 56,885,859-155,952,524 (dup) |
| 294916 | F | N/A | ≤11Y | N/A | N/A | - | + | - | - | X: 6,634,671-8,147,112 | 1.51 Mb | *HDHD1*, *STS*, *VCX*, *PNPLA4,* *MIR4767, MIR651, RPS27AP17* | Unknown heterozygous | - |
| 394804 | F | N/A | ≤5 Y | EEG abnormality | N/A | - | + | - | - | X: 60,001-17,040,343 | 17.01 Mb | 156 genes | Inherited, heterozygous | - |
| 289736 | U | N/A | N/A | Spasticity | N/A | - | - | - | - | X: 252,325-155960495 | 155.71Mb | 1,861 genes | De novo | - |

**eTable 1** Clinical features of patients with epilepsy in Xp22.31 microdeletions (continued)

| Clinical features | Sex | Nationality | Seizure onset age | Seizure type | Drugs | XLI | ID/DD | ASD | SD | Site | Size | Genes | Inheritance | Other genetic disorders |
| --- | --- | --- | --- | --- | --- | --- | --- | --- | --- | --- | --- | --- | --- | --- |
| 288698 | U | N/A | N/A | N/A | N/A | - | + | - | - | X: 162,434-58,482,384 | 58.21 Mb | 681 genes | Unknown | Chr 18: 19,075,528-19,344,069 (dup)  Chr 2: 79,969,673-80,073,702 (del) |
| 289797 | U | N/A | N/A | Generalized-onset seizure | N/A | - | - | - | - | X:6,592,473-7,843,259 | 1.20 Mb | *HDHD1*, *STS*, *VCX,* *MIR4767, RPS27AP17* | Paternally inherited | Chr 12: 14,315,734-14,538,585 (dup)  Chr 16: 52,911,795-52,975,994 (dup) |

Abbreviations: XLI: X-linked ichthyosis; DD, developmental delay; ID, intellectual disability; SD, speech disorder; ASD autism spectrum disorder; CBZ: carbamazepine; VPA: valproic acid; LEV: levetiracetam; +, indicates that the clinical feature was displayed by the corresponding patient; −, shows that the clinical feature was absent; n/a, not available; M: males; F: females; Y: year; W: week; M: month; GTC, generalized tonic-clonic seizures; CECS, childhood epilepsy with centrotemporal spikes; AE; absence epilepsy, RE: Rolandic epilepsy. Note: the phenotypes not mentioned in the DICIPHER database were regarded as “absent”, whereas they were regarded as “not available” in cohort studies, the seizure onset age was according to the “Age at last clinical assessment”.

**Table 2** Genes involved in chr X: 6451786-8138073 (hg19) microdeletion

| Gene | Gene symbol | OMIM# Gene ID | Remarks | Location (hg19) |
| --- | --- | --- | --- | --- |
| Variably charged, X Chromosome 3A | *VCX3A (VCX-A)* | 300533/51481 | Associated with intellectual disability [8, 9], but still controversial [3] | X:6533618-6535118 |
| Haloacid dehalogenase-like hydrolase domain containing 1A | *HDHD1 (PUDP)* | 306480/8226 | HDHD1dephosporylate pseudo uridine 5’-phosphate [10]; expressed at higher levels in the human brain, fetal brain and skeletal muscle [11]. | X:6667865-7148158 |
| Steroid Sulfatase | *STS* | 300747/412 | Causative for X-linked ichthyosis[12]; important for placental production of estriol during the later stages of pregnancy; encoding a steroid sulfatase which hydrolyses neuro steroids that affect membrane potential and current conductance of the neuron, controlling network excitability and seizure susceptibility [13-15] | X:6455812-8133195 |
| Variably charge X chromosome | *VCX* | 300229/26609 | Appears to be expressed only in male germ cells [25]; regulates mRNA translation and neurite outgrowth [16] | X:7842262-7844143 |
| Patatin-like phospholipase domain containing 4 | *PNPLA4* | 300102/8228 | Highly expressed in brain and skeletal muscle; involved in triglyceride hydrolysis and energy metabolism[17] | X:7898247-7927739 |
| MicroRNA 4767 | *Mir4767* | -/100616467 | - | X:7147860-7147937 |
| MicroRNA 651 | *Mir 651* | -/723779 | - | X:8126965-8127061 |
| RPS27A pseudogene 17 | *RPS27AP17* | -/392425 | - | X:6989329-6989783 |

**References**

1. Ben Khelifa, H., et al., *Xp22.3 interstitial deletion: a recognizable chromosomal abnormality encompassing VCX3A and STS genes in a patient with X-linked ichthyosis and mental retardation.* Gene, 2013. **527**(2): p. 578-83.

2. Doherty, M.J., et al., *An Xp; Yq translocation causing a novel contiguous gene syndrome in brothers with generalized epilepsy, ichthyosis, and attention deficits.* Epilepsia, 2003. **44**(12): p. 1529-35.

3. Myers, K.A., E. Simard-Tremblay, and C. Saint-Martin, *X-Linked Familial Focal Epilepsy Associated With Xp22.31 Deletion.* Pediatr Neurol, 2020. **108**: p. 113-116.

4. Olson, H., et al., *Copy number variation plays an important role in clinical epilepsy.* Ann Neurol, 2014. **75**(6): p. 943-58.

5. Malik, A., et al., *X-linked ichthyosis associated with psychosis and behavioral abnormalities: a case report.* J Med Case Rep, 2017. **11**(1): p. 267.

6. Gao, K., et al., *Large De Novo Microdeletion in Epilepsy with Intellectual and Developmental Disabilities, with a Systems Biology Analysis.* Adv Neurobiol, 2018. **21**: p. 247-266.

7. Addis, L., et al., *Identification of new risk factors for rolandic epilepsy: CNV at Xp22.31 and alterations at cholinergic synapses.* J Med Genet, 2018. **55**(9): p. 607-616.

8. Mochel, F., et al., *Normal intelligence and social interactions in a male patient despite the deletion of NLGN4X and the VCX genes.* Eur J Med Genet, 2008. **51**(1): p. 68-73.

9. Cuevas-Covarrubias, S.A. and L.M. González-Huerta, *Analysis of the VCX3A, VCX2 and VCX3B genes shows that VCX3A gene deletion is not sufficient to result in mental retardation in X-linked ichthyosis.* Br J Dermatol, 2008. **158**(3): p. 483-6.

10. Preumont, A., et al., *HDHD1, which is often deleted in X-linked ichthyosis, encodes a pseudouridine-5'-phosphatase.* Biochem J, 2010. **431**(2): p. 237-44.

11. Jenkins, C.M., et al., *Identification, cloning, expression, and purification of three novel human calcium-independent phospholipase A2 family members possessing triacylglycerol lipase and acylglycerol transacylase activities.* J Biol Chem, 2004. **279**(47): p. 48968-75.

12. Basler, E., et al., *Identification of point mutations in the steroid sulfatase gene of three patients with X-linked ichthyosis.* Am J Hum Genet, 1992. **50**(3): p. 483-91.

13. Alperin, E.S. and L.J. Shapiro, *Characterization of point mutations in patients with X-linked ichthyosis. Effects on the structure and function of the steroid sulfatase protein.* J Biol Chem, 1997. **272**(33): p. 20756-63.

14. Ballabio, A., et al., *Molecular heterogeneity of steroid sulfatase deficiency: a multicenter study on 57 unrelated patients, at DNA and protein levels.* Genomics, 1989. **4**(1): p. 36-40.

15. Kříž, L., M. Bičíková, and R. Hampl, *Roles of steroid sulfatase in brain and other tissues.* Physiol Res, 2008. **57**(5): p. 657-668.

16. Jiao, X., et al., *Modulation of neuritogenesis by a protein implicated in X-linked mental retardation.* J Neurosci, 2009. **29**(40): p. 12419-27.

17. Chen, Z., et al., *Molecular characterization, expression and chromosomal localization of porcine PNPLA3 and PNPLA4.* Biotechnol Lett, 2011. **33**(7): p. 1327-37.
